# Compilation of a self-management assessment scale for postoperative patients with aortic dissection

**DOI:** 10.1515/med-2024-0939

**Published:** 2024-04-12

**Authors:** Li-Wei Zhang, Yan-Chun Peng, Qiong Pan, Sai-Lan Li, Liang-Wan Chen, Yan-Juan Lin

**Affiliations:** Department of Nursing, Fujian Medical University Union Hospital, Fuzhou, Fujian 350001, China; Department of Cardiac Surgery Nursing, Fujian Medical University Union Hospital, Fuzhou, Fujian 350001, China; Department of Cardiac Surgery, Fujian Medical University Union Hospital, No. 29 Xinquan Road, Fuzhou, Fujian 350001, China; Department of Cardiac Surgery Nursing, Fujian Medical University Union Hospital, No. 29 Xinquan Road, Fuzhou, Fujian 350001, China

**Keywords:** aortic dissection, Delphi method, reliability and validity test, scale, self-management

## Abstract

**Objective:**

The aim of this research was to compile a self-management assessment scale for patients with aortic dissection (AD). The questionnaire is useful in making the patient aware of the need for post-operative care in order to contribute to improving the outcome and quality of life.

**Methods:**

The initial version of the “postoperative self-management assessment scale for patients with aortic dissection” was developed using the Delphi expert consultation method based on qualitative research results, consultation of self-management-related literature, reference to the existing self-management scale, and self-efficacy theory, combined with the disease characteristics of AD. By using the convenience sampling method, a total of 201 patients with AD who had undergone surgery were selected as the research participants. The initial version of the scale was used for follow-up investigation, and the scale entries were evaluated and exploratory factor analysis carried out to form the formal version of the “postoperative self-management assessment scale for patients with aortic dissection.” A total of 214 patients with AD after surgery were selected as the research participants. The formal version of the scale was used for follow-up investigation, and its reliability and validity were evaluated.

**Results:**

The formal version of the scale had 6 dimensions and 35 entries. The Cronbach’s *α* coefficient for the total scale was 0.908, the split-half reliability was 0.790, and the test–retest reliability after 2 weeks was 0.471. The content validity index of the total scale was 0.963. Exploratory factor analysis yielded six common factors, and the cumulative contribution rate of variance was 66.303%. Confirmatory factor analysis showed that except for the incremental fit index, Tucker–Lewis index, and comparative fit index >0.85, slightly lower than 0.90, *χ* ^2^/df <3, root mean square of approximation <0.08, parsimonious goodness-of-fit index, and parsimonious normed fit index >0.50; all other model fitting requirements were satisfied, indicating that the model fitting was acceptable.

**Conclusion:**

We compiled the postoperative self-management assessment scale for patients with AD, which has demonstrated excellent reliability and validity and can be used as a tool to evaluate the postoperative self-management level in patients with aortic dissection.

## 1 Introduction

Aortic dissection (AD) is one of the most critical cardiovascular diseases with a high mortality rate and elevated risk, which seriously threatens the lives of patients [1]. Surgery is a crucial treatment method for AD [1,2]. With the continuous development of surgical diagnosis and treatment, the postoperative survival rate of patients with AD has increased dramatically. According to relevant studies, the 1-year, 3-year, and 5-year survival rates of patients with AD are 92, 77, and 57%, respectively [3].

Postoperative monitoring of the disease and prevention of postoperative complications in patients with AD primarily depend on their own management. Therefore, whether discharged patients can effectively carry out self-management is of great significance for the prevention of postoperative complications and the enhancement of quality of life [4]. There is currently no evaluation tool for the postoperative self-management of patients with AD. Therefore, in this study, we begin with the definition of self-management, combined with qualitative research and related theories of patients with AD, to develop an evaluation scale for postoperative self-management of patients with AD and provide a measurement tool for a comprehensive evaluation of the postoperative self-management ability of patients with AD. The aim of this study would be useful in making the patient aware of the need for post-operative care in order to contribute to improving the outcome and quality of life.

## 2 Scale compilation

### 2.1 Preliminary determination of the scale structure

The construction of this scale was based on the self-efficacy theory proposed by Bandura [5]. The most important function of self-efficacy in self-management was to influence their own health through the regulation of self-management behavior. Bandura believed that the beginning and continuation of a particular activity or behavior were the result of the combined influence of efficacy and outcome expectations. There are four factors that influence self-efficacy: mastery experiences (such as the accumulation of experience in the results of individual daily behavior, i.e., daily life management), vicarious experiences (e.g., other ways to acquire knowledge, verbal persuasion (e.g., communicating with others), and emotion arise (e.g., symptom management, psychological coping).

According to the above theories, four sources of self-efficacy influenced the postoperative self-management behavior of patients with AD. Therefore, the initial dimensions of the postoperative self-management assessment scale for patients with AD included the following six aspects: (1) symptom management: primarily refers to the ability of the patient to cope with symptoms such as chest pain, chest tightness, palpitations, and dizziness; (2) medical staff communication: primarily refers to the communication mode and content between patients and medical staff; (3) information management: primarily refers to the patient’s mastery and acquisition of disease; (4) daily life management: primarily refers to the patient’s diet, exercise, smoking, drinking, and disease self-monitoring; (5) psychological management: primarily refers to the patient’s ability to cope with negative emotions; and (6) self-efficacy: primarily refers to the patient’s confidence when confronting disease.

### 2.2 Establish a scale entry library

#### 2.2.1 Literature research

PubMed, EBSCO, CNKI, VIP, and Wanfang databases were retrieved with the keywords of “aortic dissection” and “self-management,” [6–9], and self-management entries were collected. In addition, the chronic disease self-management scales published domestically and internationally were also retrieved, particularly the cardiovascular disease self-management scales, such as the chronic disease self-management scale [10], the self-management scale-30 [11], the diabetes self-management scale [12], the coronary heart disease self-management scale [13], the heart failure patient self-management scale [14], and so on. The entries from these scales that satisfied the self-management of patients with AD after surgery were selected as alternative entries and added to the entry library.

#### 2.2.2 Interview method

As a supplement to the literature research, semi-structured interviews were conducted with 18 postoperative patients with AD, and four themes related to the postoperative self-management of patients with AD were extracted: (1) lack of disease-related knowledge, (2) inadequate disease management behavior, (3) absence of medical communication, and (4) weak psychological state. The themes derived from the interview results were added as alternative entries to the entry library.

#### 2.2.3 Establishment of the scale entry library

Based on the results of literature research and qualitative patient interviews, the research group sorted and analyzed the collected entries, integrated those with similar meanings, deleted the duplicates, and constructed a scale entry library with 40 entries. There were 4 entries for self-efficacy, 7 for information management, 8 for symptom management, 5 entries for medical communication, 12 entries for daily life management, and 4 entries for psycho-social management.

### 2.3 Delphi expert inquiry method

One foreign expert and 12 domestic experts in cardiac surgery, cardiology, vascular surgery, clinical nursing, nursing education, and other related specialties were invited (Table 1).

**Table 1:** Basic information of experts

Item	Category	No. of people	Composition ratio (%)
Education	Doctor	7	53.84
	Master	3	23.08
	Bachelor	3	23.08
Title	Senior	6	46.15
	Vice-senior	6	46.15
	Middle	1	7.70
Years of work	10–19 years	6	46.15
	≥20 years	7	53.84
Main clinical engagement field	Cardiac surgery	7	53.84
	Cardiology	1	7.70
	Clinical nursing	3	23.08
	Nursing education	1	7.70
	Vascular surgery	1	7.70
Area	Fujian	10	76.92
	Beijing	1	7.70
	Wuhan	1	7.70
	Singapore	1	7.70

All 13 questionnaires distributed in three rounds of expert consultation were recovered. The positive coefficient of experts was 100%, the overall authority coefficient of experts (CR) was 0.981, and the Kendall’s *W* of three rounds of experts was 0.273, 0.137, and 0.429, respectively. The screening criteria were the mean score of the entry importance score ≥4 points, the coefficient of variation <0.25, and the expert recognition rate ≥75%, which were determined after the recovery of each expert correspondence questionnaire. In the first round of correspondence, 4 entries were deleted and 16 entries were added; a total of 13 entries were deleted in the second round of consultation; and in the third round of consultation, experts recognized the dimensions and entries of the scale and did not propose amendments.

The initial version of the AD patient self-management assessment scale, with 39 entries in 6 dimensions, was developed based on the screening of three rounds of consultation. The scale uses a Likert 5-level score, and 1 to 5 points indicate “none, little, sometimes, often, always.” The higher the score, the greater the patient’s self-management.

### 2.4 Preliminary investigation

To test the operability of the scale, 20 patients after AD surgery were selected for preliminary investigation to adjust the expression of entries that were hard to understand, to acquaint themselves with possible problems in the investigation process, and to prepare for the response measures in advance.

### 2.5 Formal test

In this study, patients who had the same surgery between June 2017 and June 2020 were divided into two groups using the convenience sampling method. The patients in the first group were investigated in August 2020. Inclusion criteria included: (1) diagnosed as AD by computed tomography angiography or magnetic resonance angiography, (2) age ≥18 years old, (3) patient with the ability of communication and reading comprehension, (4) patient who underwent AD surgery and was discharged from 3 months to 3 years, and (5) voluntary and informed consent to participate in this study. Exclusion criteriawere (1) patient with a history of mental illness and (2) patient with severe complications, such as heart failure or respiratory failure. With a recovery rate of 93.5%, a total of 201 valid questionnaires were collected from 215 respondents. The average age of the 201 patients with AD who participated in this survey was (54.58 ± 12.63) years.

#### 2.5.1 Entry re-screening

The entries of the scale were verified using entry analysis with the following screening criteria: (1) critical ratio method: an independent sample *t*-test was used to assess the differences in the scores of the patients between the high score and the low score groups on each entry, and entries with no significant difference were excluded. The entries with a CR value less than 3.00 were rejected from selection [15]. (2) Discrete trend method: delete the entries with a standard deviation <0.75. (3) Correlation coefficient method: delete the entries with a correlation coefficient ≤0.4. (4) Cronbach’s *α* coefficient method: calculate the Cronbach’s *α* coefficient of the total scale or each dimension, then compare the changes in the coefficients after removing a certain entry. If the Cronbach’s *α* coefficient increased significantly after the entry was removed, deletion was considered. Using the four approaches described above, the initial scale entries were screened, with four entries being deleted and 35 entries being retained.

### 2.6 Reliability and validity test of the scale

The convenience sampling method was used to conduct the second-stage survey in September 2020, and patients with AD (Part II) who underwent surgical treatment between June 2017 and June 2020 were selected as the research participants. The standard of admission remained unchanged. A total of 226 patients were interviewed in this survey, and 214 valid questionnaires were collected, with a recovery rate of 94.6%. A total of 214 patients with AD were aged between 30 and 77 years old, with an average age of (54.20 ± 11.16) years. Internal consistency analysis, split-half reliability, and test–retest reliability were used in this study to evaluate the reliability of the scale. The test–retest reliability was carried out 2 weeks after the first measurement, and 50 patients with AD were selected to retest. Pearson’s coefficient was used to evaluate the results of the two tests. The content validity of the scale was calculated by evaluating the entries with the help of an expert. The structural validity of the scale was evaluated using exploratory factor analysis and correlation analysis. Confirmatory factor analysis was carried out based on exploratory factor analysis to further verify the structure of the scale.


**Ethics approval:** This study was conducted in accordance with the declaration of Helsinki. This study was conducted with approval from the Ethics Committee of Fujian Medical University Union Hospital (2020.No.80).
**Informed consent:** A written informed consent was obtained from all participants.

## 3 Results

### 3.1 Reliability analysis

The Cronbach’s *α* coefficient of each factor of the scale was between 0.620 and 0.931, with a total scale of 0.908. The split-half reliability of each factor was between 0.493 and 0.897, with a total scale of 790 (Table 2). The test–retest reliability was 0.471 (Table 3).

**Table 2:** Internal consistency analysis of each factor and total self-management scale

Item	Cronbach’s *α* coefficient	Split-half reliability
Psychological management	0.856	0.748
Disease symptoms and management	0.931	0.897
Disease knowledge management	0.801	0.544
Self-monitoring management	0.704	0.493
Disease treatment and management	0.648	0.694
Daily life management	0.620	0.593
Total scale	0.908	0.790

**Table 3:** Test–retest reliability of each factor and total scale of self-management scale (*x* ± s)

Item	First	Second	Correlation coefficient
Psychological management	39.00 ± 6.22	40.52 ± 5.54	0.334*
Disease symptoms and management	22.34 ± 6.15	23.36 ± 6.07	0.263
Disease knowledge management	12.02 ± 3.56	12.64 ± 3.73	0.495*
Self-monitoring management	12.42 ± 3.59	12.14 ± 3.11	0.060
Disease treatment and management	15.34 ± 2.89	16.00 ± 2.99	0.557*
Daily life management	15.92 ± 2.62	16.66 ± 2.31	0.364*
Total scale	117.04 ± 17.77	121.32 ± 17.17	0.471*

Note: *indicates *P* < 0.05.

### 3.2 Validity analysis

#### 3.2.1 Content validity

The scale-level content validity index was 0.963, and the entry-level content validity index was 0.769–0.923, according to the results of the third round of expert consultation.

#### 3.2.2 Exploratory factor analysis

After screening, 35 entries were assessed for Kaiser-Meyer-Olkin measure of sampling adequacy (KMO) and Bartlett sphericity. KMO = 0.869, and the Bartlett test value was 5877.283, which reached a significant level (*P* < 0.05) and was suitable for exploratory factor analysis. Principal component analysis was used to extract common factors. Using the oblique rotation method (Kaiser standardized optimal oblique method) without limiting the extraction of common factors, the results of the first factor analysis revealed that there were nine common factors with eigenvalues >1, which could explain 76.194% of the total variation. According to the gravel map, the final number of common factors was six, and the factor analysis was repeated. The six common factors explained 66.303% of the variation, and the factor load of 35 entries was 0.520–0.934. The entries were assigned to the corresponding factors based on the size of the factor load of the rotated entries (Table A1), and the factors were named based on the content of the reintegrated entries. Finally, a formal scale with 6 dimensions and 35 entries was developed: 11 entries dealt with psychological management, 7 with disease symptom management, 5 with disease knowledge management, 4 with self-monitoring management, 4 with disease treatment management, and 4 with daily life management.

#### 3.2.3 Correlation analysis

The correlation coefficient between entries and each factor ranged between 0.451 and 0.912. The correlation coefficient between each factor ranged between 0.178 and 0.468, while the correlation coefficient between each factor and the total score was between 0.552 and 0.756 (Tables A2 and A3).

#### 3.2.4 Confirmatory factor analysis

Based on exploratory factor analysis, 214 patients from the second-stage survey were selected for confirmatory factor analysis. The results of the structural model fitting degree evaluation in this study revealed that, with the exception of incremental fit index (IFI), Tucker–Lewis index (TLI), comparative fit index (CFI) > 0.85, slightly lower than 0.90, *χ* ^2^/df < 3, root mean square of approximation (RMSEA) <0.08, parsimonious goodness-of-fit index (PGFI), and parsimonious normed fit index (PNFI) >0.50, all fulfilled the model fitting requirements, indicating that the model fitting was acceptable (Table A4).

## 4 Discussion

### 4.1 The scale is scientific and practical

The objective of this study was to develop a scale that accurately and effectively measures the self-management ability of postoperative disease treatment, daily life, and psychosocial aspects of patients with AD, to understand the self-management needs of patients, and to provide a basis for developing corresponding intervention strategies and health education.

To develop a scale, it is necessary to first establish the basic framework of the scale. In self-management, self-efficacy influences one’s health by regulating self-management behaviors [16,17]. This study is based on the theory of self-efficacy by American psychologist Bandura. By reviewing a large number of domestic and international literature on self-management research and published scales on self-management, six dimensions of the scale were preliminarily identified: disease knowledge, symptom management, healthcare communication, daily life management, psychological coping, and self-efficacy. The establishment of scale items is also an important component of the entire scale and forms the core content of the scale. The formulation of scale items should fully reflect the assessment purpose of the scale and possess scientific and applicability. In this study, through literature research and interviews with AD patients, as well as referencing items from self-management scales for cardiovascular diseases and combining them with the disease characteristics of AD itself, comprehensive information on postoperative self-management items for AD patients was obtained. The research team organized and discussed the obtained information, ultimately forming an item pool to ensure the scientific validity and applicability of scale construction. To ensure the rationality and completeness of the items, the Delphi consultation method [18] was employed during the item selection stage, inviting experts with profound theoretical research and practical experience in the field of cardiovascular specialization to evaluate and modify the scale items. Statistical methods such as critical ratio method, discrete trend method, correlation coefficient method, etc., were used to assess item sensitivity, independence, etc. Therefore, the developed scale in this study possesses a high level of scientific rigor (Table A5).

To ensure the practicability of the scale, we conducted qualitative interviews in this study with 18 patients after AD surgery so that the content of the scale could accurately reflect the needs of patients. The key concerns related to postoperative self-management in patients with AD were included in the scale entries through a literature review. In addition, the recovery rates of the two scales were 93.5 and 94.6%, respectively, and the recovery rate was high, indicating that the scale was acceptable. The initial scale eventually contained 35 entries after entry screening, and the time to complete the scale could be controlled within 20 min. Therefore, it demonstrated that the scale had good feasibility.

### 4.2 The scale has good reliability and validity

The results of this study revealed that the Cronbach’s *α* coefficient of each factor of the scale was between 0.620 and 0.931, the total scale was 0.908, the split-half reliability was between 0.493 and 0.897, and the total scale was 0.790. This demonstrated that the Cronbach’s *α* coefficient of the scale in this investigation was good, indicating that it met the requirements of measurement, and that the internal consistency of the scale in this study was good. The test–retest reliability of the scale was low. The scores of factors 1, 2, 3, 5, and 6 were higher in the second measurement than in the first measurement, which might be related to the participants receiving corresponding feedback and guidance after the first test, resulting in the influence of the retest coefficient.

In this study, a three-round expert consultation was conducted, involving 13 experts who collectively reviewed, modified, and evaluated the importance of the scale items. Based on the results of the third-round expert consultation, the content validity index of this scale was calculated to be 0.963, with content validity indices for individual items ranging from 0.769 to 0.923. This suggests that the developed scale has good content validity [19] and accurately reflects the content it intends to measure. Exploratory factor analysis results showed that the six common factors accounted for 66.303% of the total variance, indicating that these factors can effectively represent the overall structure of the scale. Furthermore, each factor was highly correlated with the total score, while correlations between factors were relatively low, indicating good internal consistency of the scale. Therefore, it can be concluded that this scale has good structural validity.

The results of confirmatory factor analysis revealed that except for the estimated values of IFI, TLI, and CFI, which were slightly lower than 0.90, the remaining indicators *χ* ^2^/df, RMSEA, PNFI, and PGFI met the requirements of reference values [20]. It also demonstrated that the scale had strong construct validity [19,21].

### 4.3 Limitations and improvement

Only patients discharged from a tertiary hospital after AD surgery were selected as the research participants in this study, which reduced the representativeness of the sample and, to a certain extent, affected the generalization of the scale. In this investigation, due to the lack of a comprehensive assessment of the “golden standard” scale for postoperative self-management of patients with AD, we were unable to test the validity of the scale using criterion-related validity. The entries of the scale must be upgraded further in future practical applications.

## 5 Conclusion

We preliminarily developed a postoperative self-management assessment scale for patients with AD with 6 dimensions and 35 entries based on the theory of self-efficacy. The results revealed that the scale has good reliability and validity and can be used to evaluate the postoperative self-management level of patients with AD.

## Appendix

**Table A1:** Structure matrix of six extracted common factors after rotation

Entry	Common factor					
	1	2	3	4	5	6
Self-management 35	0.862					
Self-management 33	0.858					
Self-management 32	0.833					
Self-management 28	0.804					
Self-management 27	0.804					
Self-management 30	0.736					
Self-management 29	0.720					
Self-management 31	0.715					
Self-management 34	0.706					
Self-management 26	0.651					
Self-management 25	0.581					
Self-management 11		0.934				
Self-management 13		0.918				
Self-management 10		0.915				
Self-management 8		0.883				
Self-management 9		0.866				
Self-management 12		0.849				
Self-management 7		0.749				
Self-management 1			0.866			
Self-management 2			0.858			
Self-management 3			0.800			
Self-management 14			0.703			
Self-management 15			0.686			
Self-management 18				0.926		
Self-management 17				0.908		
Self-management 19				0.702		
Self-management 20				0.617		
Self-management 5					0.849	
Self-management 6					0.775	
Self-management 4					0.579	
Self-management 16					0.549	
Self-management 22						0.751
Self-management 23						0.665
Self-management 24						0.601
Self-management 21						0.520

Extraction method: principal component analysis; rotation method: Kaiser standardized optimal skew method.

**Table A2:** Correlation analysis between entries and factors (*r*)

Entry	Correlation index	Entry	Correlation index
Self-management 1	0.771	Self-management 21	0.704
Self-management 2	0.72	Self-management 22	0.712
Self-management 3	0.699	Self-management 23	0.604
Self-management 4	0.703	Self-management 24	0.716
Self-management 5	0.783	Self-management 25	0.604
Self-management 6	0.743	Self-management 26	0.588
Self-management 7	0.764	Self-management 27	0.655
Self-management 8	0.861	Self-management 28	0.632
Self-management 9	0.873	Self-management 29	0.626
Self-management 10	0.822	Self-management 30	0.652
Self-management 11	0.912	Self-management 31	0.576
Self-management 12	0.757	Self-management 32	0.754
Self-management 13	0.892	Self-management 33	0.723
Self-management 14	0.777	Self-management 34	0.662
Self-management 15	0.776	Self-management 35	0.73
Self-management 16	0.451		
Self-management 17	0.797		
Self-management 18	0.785		
Self-management 19	0.661		
Self-management 20	0.676		

Note: The above were all statistically significant (*P* < 0.01).

**Table A3:** Correlation between each factor and the total scale

	Factor 1	Factor 2	Factor 3	Factor 4	Factor 5	Factor 6	Total scale score
Factor 1	1.000						
Factor 2	0.402	1.000					
Factor 3	0.348	0.313	1.000				
Factor 4	0.178	0.280	0.421	1.000			
Factor 5	0.368	0.383	0.361	0.337	1.000		
Factor 6	0.468	0.381	0.222	0.280	0.311	1.000	
Total Scale Score	0.756	0.751	0.656	0.552	0.628	0.611	1.000

Note: The above were all statistically significant (*P* < 0.01).

**Table A4:** Test results of scale model fitting

Model	*χ* ^2^	df	*χ* ^2^/df	PGFI	PNFI	IFI	TLI	CFI	RMSEA
Six dimensions	1061.636	519	2.046	0.649	0.681	0.875	0.854	0.873	0.070

## A Self-management assessment scale for aortic dissection (official version)

This questionnaire is mainly used to help us understand your confidence in self-management and how to manage yourself, so as to guide our work more effectively and let more patients benefit from it. This survey data are completely confidential and are only used as a basis for understanding the living conditions of patients and improving our work. For the following questions, please mark “√” in the corresponding form according to your actual situation (or actual feeling). There is no right or wrong answer.

**Table:**

Number	Item	Options				
**Disease knowledge management**		**Many**	**Much**	**A few**	**Seldom**	**Never**
1	My understanding of the causes, treatment, and recovery of aortic dissection					
2	I will take the initiative to obtain information about aortic dissection through the Internet, reading books, and other ways					
3	I can identify the reliability of the disease information obtained					
4	I will discuss any issues related to my condition with the medical staff					
5	I get what I want from the medical staff					
**Disease treatment and management**		**Always**	**Often**	**Sometimes**	**Seldom**	**Never**
6	If I take medication, I know what it does, how to use it, how much to use it, the side effects, and what to look out for					
7	I know the importance of aortic dissection review					
8	I will follow the doctor’s advice and go to the hospital regularly					
9	If I take medicine, I will adhere to the doctor’s advice on time and dosage					
**Disease symptom management**						
10	When adverse drug reactions occur, I can communicate with the doctor in time and adjust the medication according to the doctor’s advice					
11	When adverse drug reactions occur, I can communicate with the doctor in time and adjust the medication according to the doctor’s advice					
12	When sudden chest, lower back, or abdominal pain occurs, I seek immediate medical attention or help					
13	I will deal with my body pain correctly and try not to interfere with my normal life					
14	If numbness or weakness occurs in my limbs, I seek medical attention or help					
15	When I feel tired, have palpitations, or have shortness of breath, I rest immediately					
16	If the cough worsens during manual labor or lying flat at night, I will seek medical attention or seek help in time					
**Self-monitoring management**		**Always**	**Often**	**Sometimes**	**Seldom**	**Never**
17	I monitor my pulse every day and record it					
18	I monitor my blood pressure every day and record it					
19	I monitor my blood lipids regularly and record them					
20	I can monitor my weight regularly and keep it in the normal range					
**Daily life management**		**Always**	**Often**	**Sometimes**	**Seldom**	**Never**
21	I eat foods that are low in cholesterol, high in vitamins, high in fiber, and high in protein					
22	I can work and rest regularly, don’t stay up late, and ensure a good sleep					
23	I can keep my bowels open and avoid straining					
24	I can choose appropriate exercise items, exercise intensity, and exercise time according to my own situation or the advice of medical staff, so as to avoid recurrence of aortic dissection caused by high blood pressure					
**Psychological management**		**Always**	**Often**	**Sometimes**	**Seldom**	**Never**
25	I can still do the things or hobbies I love					
26	I can actively participate in rehabilitation exercises related to illness					
27	I am able to stay positive and try to look on the bright side of life					
28	When I am in a bad mood, I will actively seek some ways to adjust my mood (such as walking, listening to music, watching TV, etc.)					
29	I can handle the relationship with my family and friends well, and communicate with them proactively when I encounter troubles and anxiety					
30	I can accept the fact that I have this disease					
31	I still have plans for the rest of my life					
32	I was able to set disease management goals and establish behavior plans					
33	I have great faith in the treatment and recovery of the disease					
34	I have the confidence to change the lifestyle habits that are not conducive to recovery from the disease (according to the previous)					
35	I am confident that I can control the bad emotions caused by the disease					
